# Abnormal lateralization of fine motor actions in Tourette syndrome persists into adulthood

**DOI:** 10.1371/journal.pone.0180812

**Published:** 2017-07-14

**Authors:** D. Martino, C. Delorme, E. Pelosin, A. Hartmann, Y. Worbe, L. Avanzino

**Affiliations:** 1 Department of Clinical Neurosciences, University of Calgary, Calgary, AB, Canada; 2 UMR S 975, CNRS UMR 7225, ICM, Sorbonne Universités, UPMC University Paris 06, Paris, France; 3 Department of Neurology, Groupe Hospitalier Pitié-Salpêtrière, Assistance Publique-Hôpitaux de Paris, 47–83 boulevard de l'Hôpital, Paris, France, and French National Reference Centre for Gilles de la Tourette Syndrome, Groupe Hospitalier Pitié-Salpêtrière, Paris, France; 4 Department of Neuroscience, Rehabilitation, Ophthalmology, Genetics and Maternal Child Health, University of Genoa Genoa, Italy; 5 Department of Neurophysiology, Saint-Antoine Hospital, Paris, France; 6 Section of Human Physiology and Centro Polifunzionale di Scienze Motorie, Department of Experimental Medicine, University of Genoa Genoa, Italy; University of Ottawa, CANADA

## Abstract

Youth with Tourette syndrome (TS) exhibit, compared to healthy, abnormal ability to lateralize digital sequential tasks. It is unknown whether this trait is related to inter-hemispheric connections, and whether it is preserved or lost in patients with TS persisting through adult life. We studied 13 adult TS patients and 15 age-matched healthy volunteers. All participants undertook: 1) a finger opposition task, performed with the right hand (RH) only or with both hands, using a sensor-engineered glove in synchrony with a metronome at 2 Hz; we calculated a lateralization index [(single RH–bimanual RH)/single RH X 100) for percentage of correct movements (%CORR); 2) MRI-based diffusion tensor imaging and probabilistic tractography of inter-hemispheric corpus callosum (CC) connections between supplementary motor areas (SMA) and primary motor cortices (M1). We confirmed a significant increase in the %CORR in RH in the bimanual vs. single task in TS patients (p<0.001), coupled to an abnormal ability to lateralize finger movements (significantly lower lateralization index for %CORR in TS patients, p = 0.04). The %CORR lateralization index correlated positively with tic severity measured with the Yale Global Tic Severity Scale (R = 0.55;p = 0.04). We detected a significantly higher fractional anisotropy (FA) in both the M1-M1 (*p* = 0.036) and the SMA-SMA (*p* = 0.018) callosal fibre tracts in TS patients. In healthy subjects, the %CORR lateralization index correlated positively with fractional anisotropy of SMA-SMA fibre tracts (R = 0.63, p = 0.02); this correlation was not significant in TS patients. TS patients exhibited an abnormal ability to lateralize finger movements in sequential tasks, which increased in accuracy when the task was performed bimanually. This abnormality persists throughout different age periods and appears dissociated from the transcallosal connectivity of motor cortical regions. The altered interhemispheric transfer of motor abilities in TS may be the result of compensatory processes linked to self-regulation of motor control.

## Introduction

Tourette syndrome (TS) is a childhood-onset neurodevelopmental disorder defined by persistent motor and vocal tics [1]. TS co-occurs with other neurodevelopmental disorders, most commonly attention deficit hyperactivity (ADHD) and obsessive-compulsive (OCD) disorders, in about 85% of patients [2]. In addition to tics and behavioural disturbances, there is evidence in TS of abnormalities in the execution of tasks requiring manual dexterity, e.g. the Purdue Pegboard test [3]. Movement lateralization influences the proficiency in executing bimanual movements and shifting from unimanual to bimanual motor tasks; such motor abilities have a profound impact on activities of daily living and quality of life. We previously documented that voluntary finger movements are less lateralized in children with “pure” TS (i.e. without clinically relevant behavioural comorbidities), leading to a better performance during bimanual tasks, which suggests atypical development of sensorimotor integration, movement lateralization and bimanual coordination of fine motion in this patient population [4].

Some aspects of this abnormal ability to lateralize fine manual tasks remain poorly understood. First, we still ignore whether this trait persists in TS patients who continue to exhibit tics throughout adulthood, and whether it correlates with tic severity. Second, we have limited information on the neuroanatomical substrate of this motor abnormality. The microstructure of interhemispheric projections, in particular those of the corpus callosum (CC), is extremely important for interhemispheric information exchange, movement lateralization and bimanual coordination [5,6]. Diffusion tensor imaging (DTI) studies showed an association between the development of the CC microstructure and bimanual coordination in healthy adolescents [7]. In young and older adults, bimanual coordination was significantly related to the microstructural properties of the more anterior sub-regions of the CC, including the premotor/supplementary and primary motor [8,9].

Several imaging studies highlighted differences of CC size and microstructural changes (fractional anisotropy) of motor sub-regions of the CC between TS patients and typically developing individuals of different ages, which seem independent from comorbidities [10–16]. The relationship between interhemispheric transfer and microstructure of callosal sub-regions connecting motor cortical regions was found to be abnormal in adult TS patients [11]. This suggests the possibility of a different morphological substrate of bimanual coordination in individuals with TS.

In order to advance our understanding of movement lateralization and bimanual coordination in this condition, we evaluated: i) whether patients with TS persisting in adulthood exhibit the same motor lateralization abnormality as younger TS patients; ii) whether the performance on a motor task involving finger motor sequences is related to microstructural changes of motor sub-regions of the CC in adults with TS. Given that adult patients with TS are likely to represent a more severe subgroup of individuals with this condition, we predicted to confirm the presence of the motor lateralization abnormalities previously detected in paediatric patients. We also predicted to detect a correlation between kinematic performance and the microstructure of callosal sub-regions connecting motor cortical regions.

## Materials and methods

### Subjects

Patients were recruited from the Gilles de la Tourette Syndrome Reference Centre at the Pitié-Salpêtrière Hospital in Paris, France. Thirteen patients with TS (7 males; mean ± SD age 33 ± 10.3 years) participated in the study. Inclusion criteria for patients were: i) age >18 years; ii) having a confirmed diagnosis of TS according to the Diagnostic and Statistical Manual of Mental Disorders-5 criteria [17]. Exclusion criteria comprised: i) co-occurrence of Axis I psychiatric disorders, established by the Mini International Neuropsychiatry Inventory [18], with the exception of obsessive-compulsive disorder; co-occurrence of autistic spectrum disorder, substance abuse aside from nicotine, current major depressive episode, current or past diagnosis of psychotic disorder; ii) any neurologic disorder other than tics; iii) visual or hearing impairment; iv) severe orthopaedic problems of the upper limb. Thirteen age- and sex-matched healthy subjects (HS, 6 males; mean age 32.8 ± 6.8 years) were recruited as control subjects from hospital staff or patients’ spouses or friends. Exclusion criteria were the same as for TS patients, plus i) a personal history of tics, and ii) any concomitant treatment except for oral contraceptives. All participants were right-handed; right hand dominance was confirmed using the Edinburgh Handedness Inventory [19]: Edinburgh Handedness inventory scores ranged from 90 to 100, indicating a strictly right-hand dominance. Demographic and clinical information for patients with TS are reported in Table 1. Three TS patients were on treatment with antipsychotic drugs (aripiprazole, risperidone, pimozide), one with citalopram, and one with clonazepam; in each of these patients, the medication dose had been stable for at least 4 weeks. Tic severity was assessed using the Yale Global Tic Severity Scale (YGTSS), 0–50 severity sub-score [20]. The local ethics committee (Pitié-Salpêtrière Hospital) approved the study and every participant gave informed written consent for participation. The Ethics committee project' number is INSERM C11-34, CPP 97/12. The experimental procedures were carried out in agreement with legal requirements and international norms (Declaration of Helsinki, 1964).

**Table 1:** Main demographic and clinical data of patients with Tourette syndrome. OCBs: obsessive-compulsive behaviour. OCD: obsessive-compulsive disorder.

Patient	Age (years)	Sex	Edinburgh handedness score	Age at onset (years)	Yale Global Tic Severity Scale (severity subscore / 50)	Yale Global Tic Severity Scale (total score/ 100)	Presence of obsessive-compulsive symptoms	Current pharmacological treatment
**1**	42	F	100	6	10	20	OCBs	Clonazepam
**2**	29	F	100	6	12	12	No	Aripiprazole
**3**	38	M	100	7	18	38	OCD	Citalopram
**4**	24	M	90	6	14	14	No	None
**5**	30	M	100	8	12	32	OCBs	None
**6**	33	M	90	6	8	8	No	None
**7**	22	M	100	10	14	24	No	Risperidone
**8**	24	F	100	6	22	32	No	None
**9**	30	F	100	7	19	29	No	None
**10**	33	M	90	6	27	47	No	Pimozide
**11**	40	F	100	8	22	32	No	None
**12**	60	F	100	5	15	35	No	None
**13**	24	M	90	6	14	24	No	None

### Motor task

**Fig 1:**
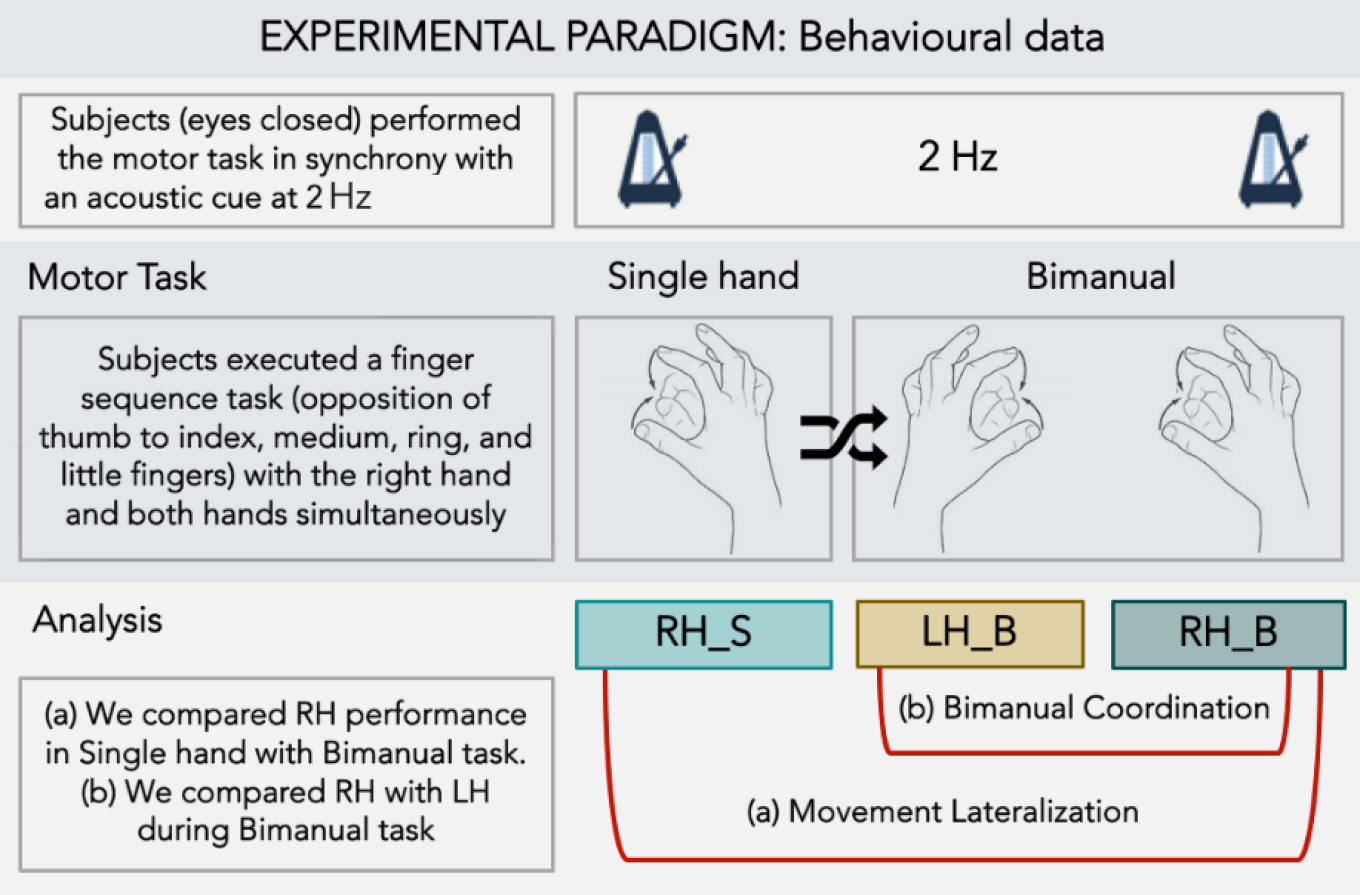
Experimental paradigm. Subject performed with eyes closed a finger opposition movement task (opposition of thumb to index, medium, ring and little fingers), following an acoustic cue set at 2 Hz. The task was executed with right hand only (single hand task; RH-S) or both the right (RH-B) and left (LH-B) hands simultaneously (bimanual task). The analysis was planned to study for movement lateralization and bimanual coordination.

Subjects sat in a comfortable chair and wore a sensor-engineered glove (Glove Analyzer System, eTT s.r.l., Genova, Italy) on both hands. Subjects were shown the finger sequence task (opposition of thumb to index, medium, ring, and little fingers) only one time and then, with eyes closed, they were asked to perform it in synchrony with an acoustic cue paced at 2 Hz. Subjects performed the task using only the right hand (single hand task) and both hands simultaneously (bimanual task); the order in which the two versions of the task (task condition) were performed was pseudo-randomized. For each task condition, we conducted a 45-seconds trial followed by a 1-minute rest (Fig 1).

We evaluated the following kinematic parameters of the finger motor sequence: *touch duration* (TD), defined as the contact time between the thumb and another finger; *inter-tapping interval* (ITI), defined as the time between the end of the contact between the thumb and another finger and the beginning of the contact between thumb and the adjacent finger; and *number of correct movements* expressed as a percentage of the total number of movements requested by the task (%CORR). Kinematic parameters were analysed to explore: (1) movement lateralization, by comparing the right hand performance on the single-hand task to the right hand performance on the bimanual task; (2) bimanual coordination, by comparing the performance of the right hand to the performance of the left hand during the bimanual task. We described the efficiency of movement lateralization by calculating the *lateralization index* (i.e. [right hand performance on the bimanual task–right hand performance on the single hand task] / right hand performance on the single hand task x 100) for all kinematic parameters. We also described the efficiency of bimanual coordination by calculating the *bimanual index* (i.e. [left hand performance on the bimanual task–right hand performance on the bimanual task] / right hand performance on the bimanual task x 100) for all kinematic parameters.

### Magnetic resonance tractography analysis

#### Image acquisition

Images were acquired on a 3T Siemens Trio MRI scanner (body coil excitation, 12-channel receive phased-array head coil). Anatomical scans were acquired using sagittal 3D T1-weighted magnetization prepared rapid acquisition gradient echo. The characteristics of diffusion weighted scans were as follow: echo time (TE): 87 ms; repetition time (TR): 12 s; 65 slices; matrix: 128x128; voxel size: 2x2x2 mm^3^; partial Fourier factor: 6/8; grappa factor: 2; read bandwidth: 1502 Hz/pixel; flip angle: 9°. Diffusion weighting was performed along 50 directions with a b-value of 1000 sec·mm^-2^. A reference image with no diffusion weighting was also obtained. Patients were asked to suppress their tics during the acquisition in order to avoid movement artefacts.

#### Image processing

Images pre-processing was carried out using FSL toolbox from the FMRIB Software Library. Diffusion images were corrected for eddy current artefacts, and fractional anisotropy (FA) maps were generated using FDT (FMRIB’s Diffusion Toolbox). The analytical Q-ball model was applied to estimate the local underlying orientation distribution function (ODF) using a spherical harmonics order 6 and a regularization factor equal to 0.006 [21]. The high-resolution 3D T1 volume was realigned to the diffusion data.

The probabilistic distributions of the fibre orientations were calculated at each voxel using a constrained spherical deconvolution (CSD) model with the MRtrix software [22]. MRtrix uses an algorithm than combines CSD with probabilistic streamlines tractography [23]. CSD can model multiple fibre populations within a single voxel and overcomes the crossing fibre limitations inherent to diffusion tensor imaging.

Whole-brain probabilistic tractography was performed in the native space of each subject by specifying the white matter map provided by the segmentation of the anatomical T1-weighted image as the seed and the whole brain as target, with the following parameters: step size = 0.1mm, number of track = 500000. Streamlines were generated by randomly seeding throughout the whole brain until the total number of 500000 streamlines had been generated.

**Fig 2:**
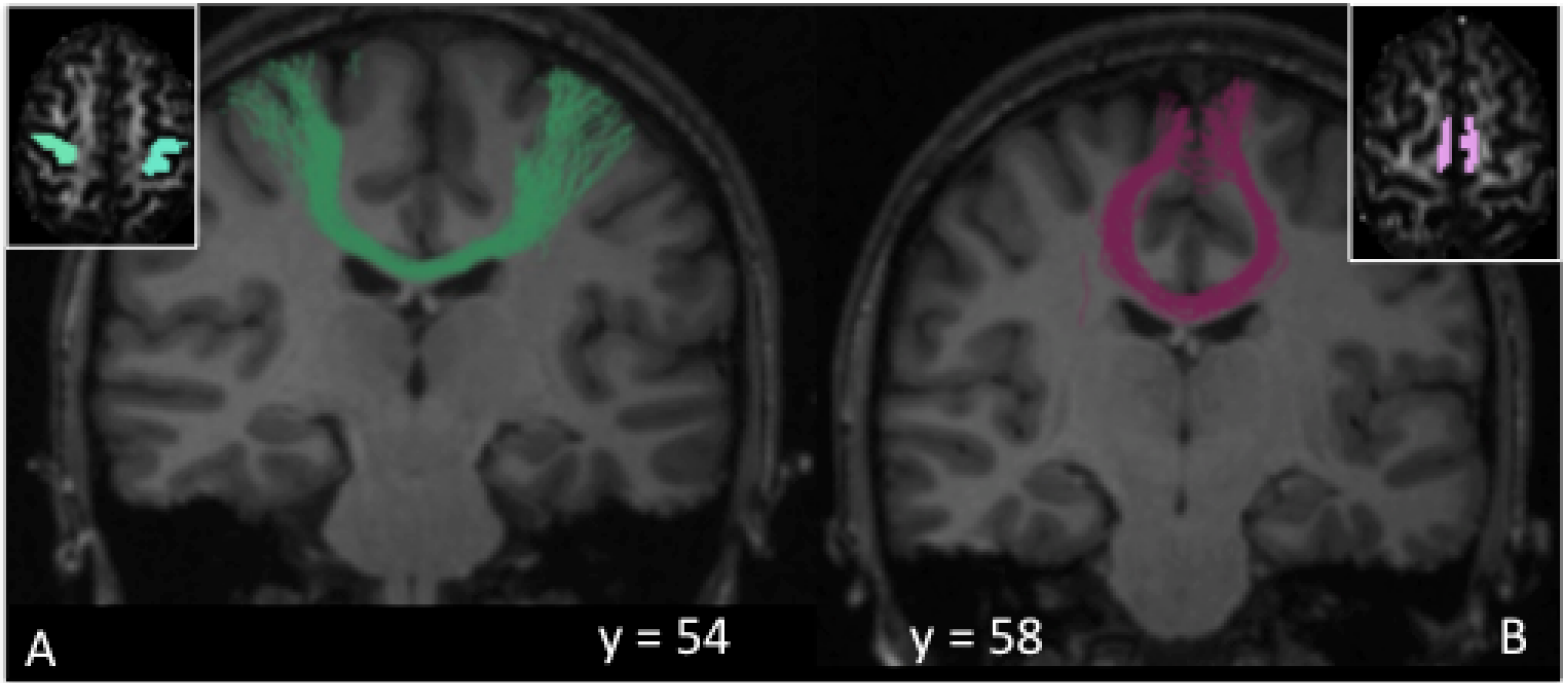
Regions of interest-seeded tractography. Fig 2A shows the callosal fibre tracts interconnecting the primary motor area (M1) of the two hemispheres, whereas Fig 2B shows the callosal fibre tracts interconnecting of the supplementary motor area (SMA) of the two hemispheres.

Regions of interest (ROI) masks were created manually in each participant’s diffusion space using cortical landmarks on the co-registered T1-weighted structural image as previously described [24]. The primary motor cortex (M1) mask was chosen as the motor cortex and underlying white matter on the two axial slices where the “hand knob” region was best identified [25]. The supplementary motor area (SMA) was anatomically defined as the medial cortex caudal to a line drawn through the anterior commissure perpendicular to the anterior commissure-posterior commissure line [26]. These ROI were used to reconstruct tracts of interest. We reconstructed tracts between the two M1 (M1-M1 tract) (Fig 2A) and two SMA (SMA-SMA tract) (Fig 2B) regions. FA was extracted from each tract of interest in every subject.

### Statistical analysis

#### Power analysis

One of the aims of the present study is to assess whether the differences between adult TS patients and age- and gender-matched control subjects are similar to those previously observed in paediatric groups. Therefore, we based our power analysis on the group differences observed in paediatric groups using the same methodology [4]. In this previous study, sample sizes of n = 11 (patients) and n = 13 (control participants) yielded on 2-sided tests statistically significant between-group differences at a *p*<0.001 level for TD and ITI and at a *p* = 0.011 level for %CORR, with corresponding power of 97%, 93% and 95%, respectively. In that study, we also detected significant differences between single-hand and bimanual tasks within control participants for TD, ITI and %CORR at significance levels of *p* = 0.005, *p* = 0.01 and *p* = 0.001, respectively. On the basis of these assumptions, we anticipated that a sample size of 13 subjects per group would be adequate to show statistically significant differences between groups on the three kinematic parameters explored.

#### Demographic data

Gender differences between groups (TS, HS) were assessed by χ^2^ test. Differences between groups for age and Edinburgh handedness score were analysed by unpaired *t* tests.

#### Behavioural data

To analyse movement lateralization, right hand parameters on single hand and bimanual tasks were compared using an analysis of variance for repeated measures (RM-ANOVA) with the variable GROUP (TS and HS) as between-subject factor and TASK (single hand, bimanual) as within-subject factor. An unpaired *t* test was used to compare the movement lateralization indexes between groups. To analyse bimanual coordination, right and left hand parameters on the bimanual task were compared using RM-ANOVA with the variable GROUP as between-subject factor and SIDE (right, left) as within-subject factor. An unpaired *t* test was used to compare the bimanual coordination indexes between groups. When RM-ANOVA gave a significant result (*p* < .05), post hoc analysis was performed using *t* tests and applying the Bonferroni correction for multiple comparisons where necessary.

We performed correlation analyses between clinical data (tic severity measured with the Yale Global Tic Severity Scale [YGTSS], range 0–50) and outcome measures of the task (movement lateralization and bimanual coordination indexes) calculating Pearson’s correlation coefficient.

#### Neuroimaging data

Mean FA measures from M1-M1 and SMA-SMA transcallosal fibre tracks were compared between the two groups using univariate ANOVA (separately for each tract) with age as a covariate of non-interest.

We also performed correlation analyses between FA values of M1-M1 callosal fibre tracts and SMA-SMA callosal fibre tracts for each hemisphere, and outcome measures of the motor task (movement lateralization and bimanual coordination indexes) using Pearson’s correlation test. These correlation analyses were performed separately for the TS patients and HS groups. All statistical analyses were performed with SPSS 22.0.

## Results

The two groups were age- (TS patients, mean age 33 years; healthy subjects, mean age 32.8 years, *p*>0.05) and gender -matched (p>0.05). No between-group differences were found with respect to Edinburgh handedness scores (TS patients, mean score 96.92; healthy subjects, mean score 96.15, *p*>0.05).

### Kinematic analysis

#### Movement lateralization

**Fig 3:**
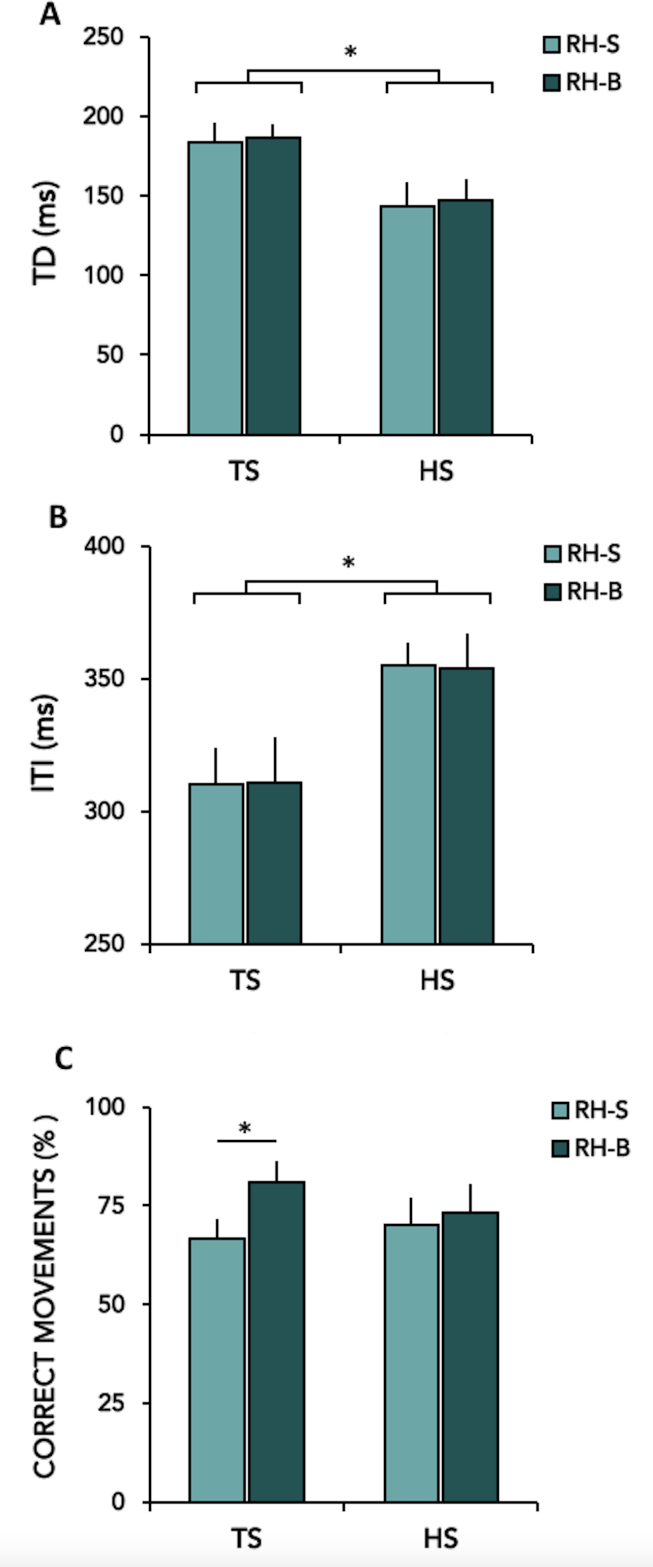
Motor behaviour parameters in healthy subjects (HS) and patients with Gilles de la Tourette syndrome (TS) during the execution of single- hand finger sequence with the right hand (RH-S) and during bimanual finger sequence with the right (RH-B) hand. A–B: ordinate, value of TD and ITI in milliseconds; C: ordinate, % of CORRECT MOVEMENTS in percentage on total movements. Means ± standard errors of mean (SEM) of data are shown. Asterisks indicate when statistical analysis showed a significant difference (* p < 0.05, ** p < 0.01).

**Fig 4:**
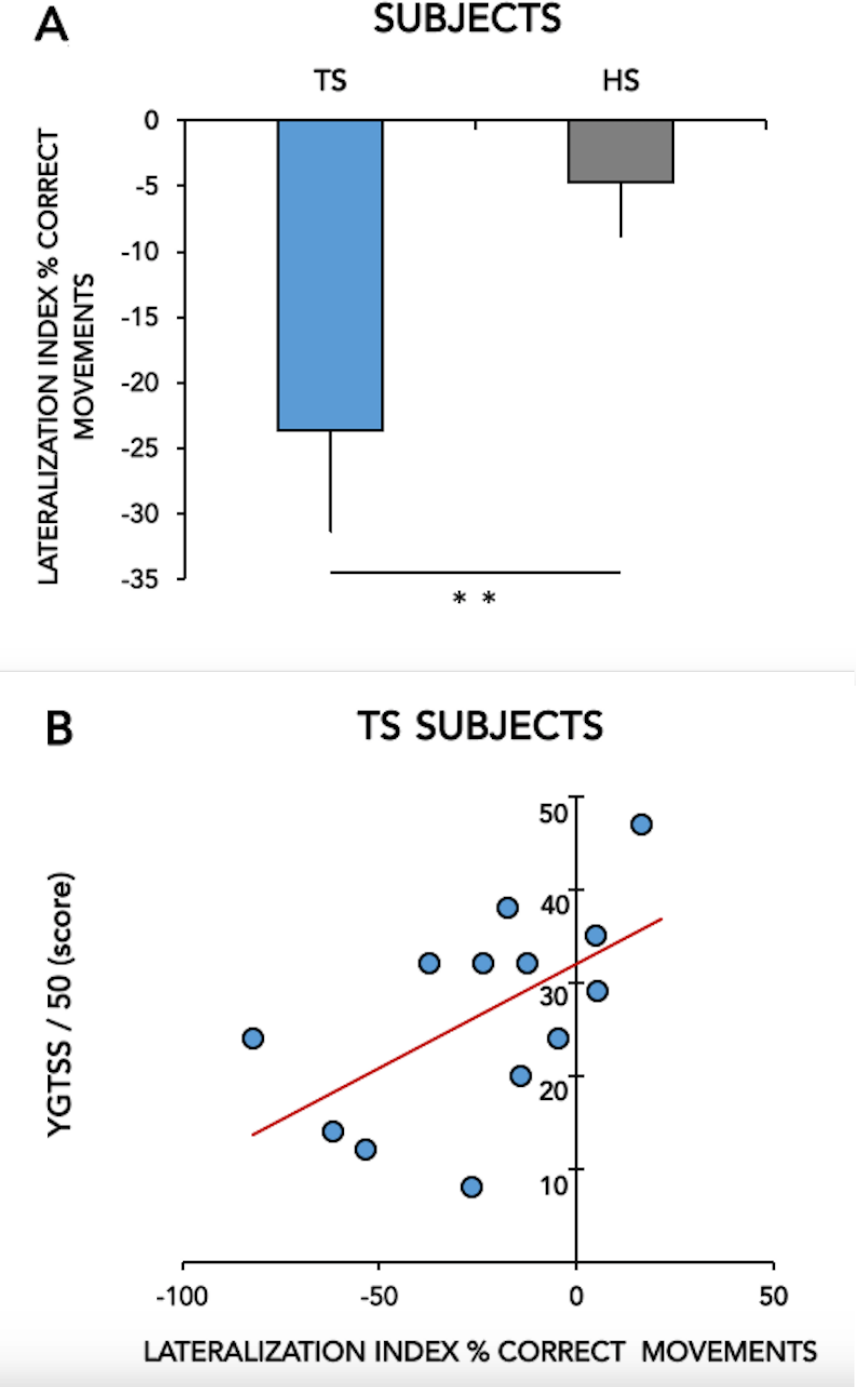
Fig 4A shows the lateralization index of the % CORRECT MOVEMENTS in healthy subjects (HS) and patients with Gilles de la Tourette syndrome (TS), calculated as [% CORR MOV RH-B–% CORR MOV RH-S / % CORR MOV RH-S x 100]. Means ± standard errors of mean (SEM) of data are shown. Asterisks indicate when statistical analysis showed a significant difference (* p < 0.05, ** p < 0.01). Fig 4B shows the correlation between the lateralization index of the % CORRECT MOVEMENTS (X-axis) and the YGTSS/50 score (Y-axis) in patients with Gilles de la Tourette syndrome (TS).

Behavioural data are summarised in Fig 3 and Fig 4A. Comparing right-hand performance in single-hand task *vs* bimanual task in TS and healthy subjects, we observed a significant effect of GROUP for TD (F(1,24) = 5.58; p = 0.027) and ITI (F(1,24) = 5.93; p = 0.023). Post hoc analysis showed that, while performing finger opposition movements with the dominant hand in single-hand or bimanual tasks, TS patients devoted a longer period of time to finger contact (TD) (Fig 3A) and less time to finger motion between contacts (ITI) (Fig 3B) compared to control subjects (TD: TS vs HS, p = 0.027; ITI: TS vs HS, p = 0.023). The lateralization index for TD, ITI and TD/ITI was not different between groups.

For %CORR, a significant GROUP*TASK interaction was found (F(1,24) = 5.44; p = 0.028). Post hoc analysis revealed a significant increase in the number of correct movements (%CORR) performed with the right hand in the bimanual vs. single hand task in TS patients (p<0.001) (Fig 3C). Accordingly, lateralization index was significantly different between the two groups (significantly lower lateralization index for %CORR in TS patients, p = 0.04) (Fig 4A).

#### Bimanual coordination

We did not detect any significant difference between TS patients and HS on the explored kinematic parameters. However, a main effect of SIDE was found for TD, ITI and TD/ITI: all subjects showed an increased TD (SIDE, F(1,24) = 26.09; p<0.001) and a decreased ITI (SIDE, F(1,24) = 45.96; p<0.001) with the left hand compared to the right hand. There was no difference between and within groups (p always >0.05) for the %CORR parameter. The bimanual index for TD, ITI, and %CORR did not differ between groups (all *p* values >0.05).

#### Clinical-kinematic correlation

When we analysed the correlation between kinematic parameters and tic severity, we identified a significant positive correlation between the %CORR lateralization index and the severity sub-score of the YGTSS (r = 0.56, *p* = 0.04; Fig 4B).

### Tractographic analysis of transcallosal fibre tracks

In TS patients compared to HS, we found a significantly higher FA in both the M1-M1 (F(1,25) = 4.888, p = 0.036; mean FA ± SD: 0.551 ± 0.116 in TS patients and 0.503 ± 0.023 in HS) and the SMA-SMA (F(1,25) = 6.375, p = 0.018;, mean FA ± SD: 0.551 ± 0.109 in TS patients and 0.499 ± 0.018 in HS) transcallosal tracts. There were no differences in the ROI mask size between groups in both hemispheres (all *p* values > 0.524), which could have potentially confounded our results.

#### Clinical-tractographic correlation

We did not observe a significant correlation between FA values in either of the transcallosal tracts and severity of tics measured by YGTSS severity sub-score (*p* value > 0. 45).

#### Kinematic-tractographic correlation

**Fig 5:**
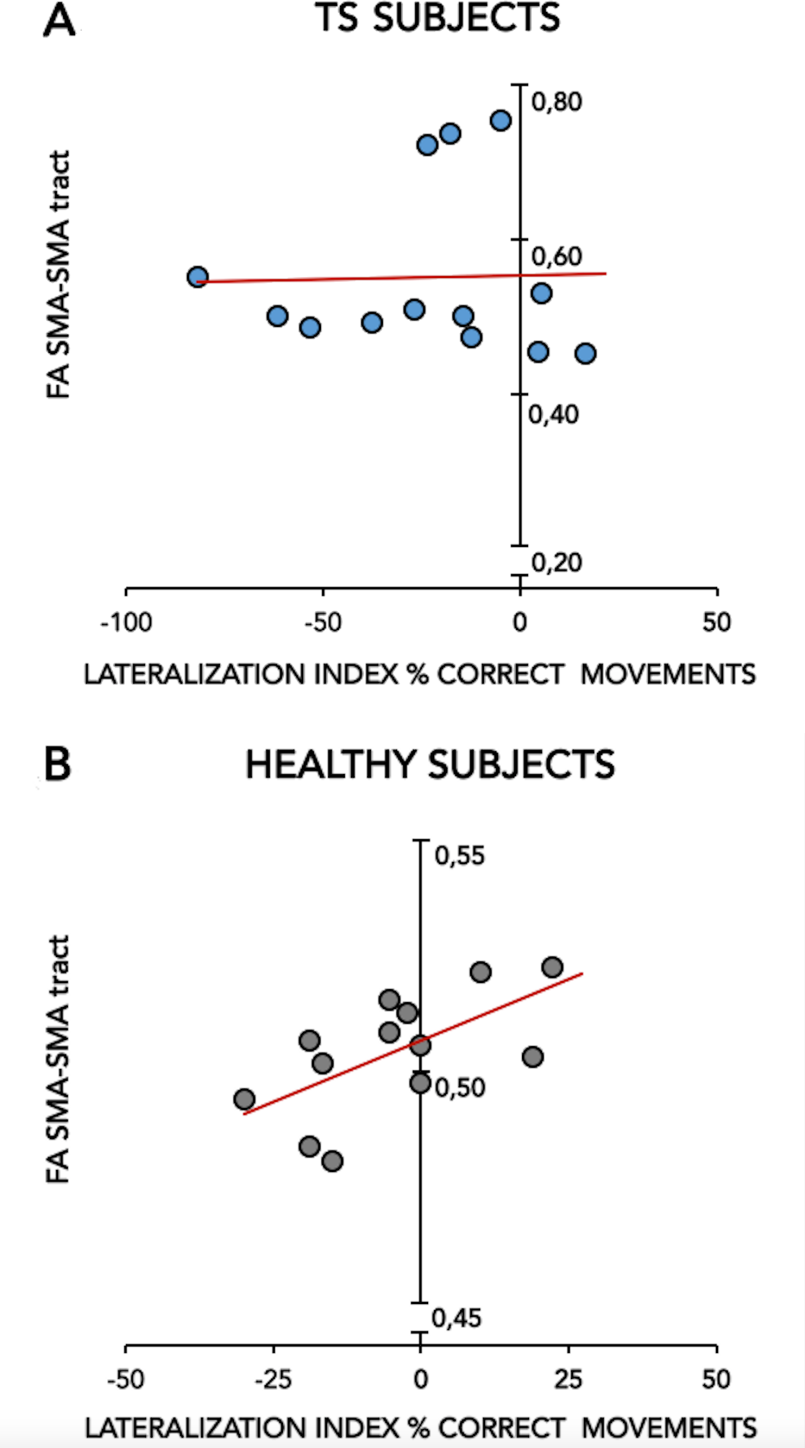
Correlation between the lateralization index of the % CORRECT MOVEMENTS (X-axis) and the FA SMA-SMA tract (Y-axis) in patients with Gilles de la Tourette syndrome (TS) (Fig 5A) and healthy subjects (HS) (Fig 5B).

We detected a significant positive correlation between the %CORR lateralization index and the FA of SMA-SMA callosal fibre tracts (r = 0.63, *p* = 0.02; Fig 5A) in healthy subjects; this correlation was absent in GTS patients (r = 0.028, *p* = 0.92; Fig 5B). TD and ITI lateralization indices and all bimanual coordination indices did not correlate significantly with any FA value (all *p* >0.05).

## Discussion

The performance on the finger sequential task in our adult patients with TS showed both similarities and differences to that previously reported in children with TS [4]. Like paediatric patients, our adult TS patients exhibited longer touch duration (likely representing the combination of *sensory* and *motor preparation* phases within the sequence) and shorter inter-tapping interval (representing a pure *motor* phase) in the dominant hand performance. This executional pattern, which could reflect abnormal processing of sensory information during the motor preparation phase, appears therefore to be constantly present in patients with TS from childhood to adulthood. However, this pattern was not associated with a reduced accuracy on right hand performance on the single-hand task (%CORR) in TS adults as opposed to TS children, probably because of increased effectiveness of motor control acquired with age in both patients and control subjects. In line with this, we did not observe any significant loss of accuracy on the bimanual task compared with the single hand in either group, whereas we had observed this in the healthy children group in our previous study.

Regarding movement lateralization, TS adults gained in right hand accuracy on the bimanual task with respect to the single-hand task. This difference in accuracy between the two types of task was statistically significant for TS patients, whereas accuracy did not differ significantly between the two tasks in healthy adults. This pattern is similar to that observed in children [4] and is reflected by a lower %CORR lateralization index. This finding suggests an abnormal ability to lateralize movement, with a “gain of function” in terms of accuracy of right hand performance when performing fine manual sequences bimanually as opposed to a single-hand modality. This trait is stable in TS persisting in adult life and likely represents an epiphenomenon of compensatory mechanisms, given that the %CORR lateralization index has a strong, positive correlation to tic severity. Given the relatively small sample size in our study, we acknowledge that independent replication in other patient groups would consolidate these findings.

In the tractography analysis, our group of adults with TS exhibited higher FA values of the fibre tracts within the motor sub-regions of the CC, interconnecting the primary motor and supplementary motor cortical regions of the two hemispheres. In line with our findings, earlier studies of interhemispheric connectivity in TS patients across different age groups reported a larger CC size in TS adults compared to age-matched control subjects, whereas CC size in TS children was smaller than in typically developing youth [11,12]. The same earlier studies also detected a positive correlation between CC size and tics severity, probably in relationship to defective self-regulatory mechanisms in the anterior frontal regions in patients with TS persisting in adulthood [11]. Conversely, we could not observe any correlation between FA values in the motor sub-regions of the CC and tic severity. A possible explanation for this lack of correlation is that the higher FA values in the motor sub-regions of the CC represents a developmental trait of patients with persistent TS that is unlikely to influence, or be influenced by, a widely short-term variable state measure like tic severity [27].

Findings on CC microstructural organisation in TS are not uniform in the literature. For instance, other groups have reported both a decreased FA associated with increased radial diffusivity throughout all callosal sub-regions [14] and a lack of difference between patients and control subjects [15] in adult TS patients without comorbidities. The discrepancy between the studies exploring CC microstructure could be due to methodological differences. For instance, in this study we used ROI of the primary motor and supplementary motor areas resulting in FA values in M1-M1 and SMA-SMA tracts, whereas Baumer et al. [15] directly used the CC as ROI using manual delineation and compared FA within this ROI between patients and controls. Compared to Neuner et al. [14], who performed voxel-wise statistical analyses to compare FA skeletons of white matter tracts across the whole brain between TS patients and controls, here we used univariate ANOVA comparison of FA values in the aforementioned tracts between both groups. Finally, the strength of magnetic field (3T vs 1.5 T) used could also have influenced this discrepancy across studies.

What could be the link between the kinematic and the anatomical traits observed in our patients with persisting TS? In our healthy control group, the %CORR lateralization index, which expresses how lateralized the performance accuracy of our finger sequential task is, was positively correlated to the degree of organisation of callosal tracts interconnecting the supplementary motor area of the two hemispheres. This expected finding is in keeping with the established relationship between interhemispheric connectivity and ability to lateralize fine bimanual tasks in healthy individuals [8]. We found that this relationship was lost in patients with TS, which leads to two main considerations. Firstly, this lack of correlation indicates a *dissociation between interhemispheric connectivity and lateralization of fine manual tasks* in TS. This is consistent with a previous study by Baumer et al. [15] who found absence of correlation between FA in the motor region of the CC and left-to-right interhemispheric inhibition measured with paired-pulse transcranial magnetic stimulation in adults with TS, whereas a correlation was detected in their age-matched control population. This dissociation stands out with even greater evidence from our study, given that our TS patients showed lower %CORR lateralization indices compared to control subjects despite higher FA values in the motor region of the CC. A second consideration relates to the anatomical substrate of the reduced %CORR lateralization index in TS adults. Although our study focused exclusively on motor transcallosal connectivity and did not analyse functional activity of brain regions, it is possible that the abnormal ability to lateralize fine manual tasks represents a compensatory trait associated with brain regions involved in self-regulation of motor control in TS. In line with this, a functional MRI study [28] demonstrated that young patients with TS, despite equiperformance with typically developing youth, over-recruit the contralateral premotor and prefrontal areas as well as the ipsilateral inferior parietal lobule during simple index finger tapping using the non-dominant hand, and that this over-recruitment is positively correlated to tic severity. We hypothesize that the reduced %CORR lateralization index is epiphenomenal to the failure of compensatory mechanisms, possibly involving regions of the frontal cortex important for motor control, that were initially put in place to homeostatically preserve and adapt motor control to the pathological release of unwanted movements, i.e. tics. In this view, it is possible that the higher FA values represent a structural correlate of this failed attempt to preserve lateralized motor control. Consistently, Buse et al. [29] reported that the callosal sub-region 3, which includes the fibre tracts examined in our study, exhibits progressive growth over time during development in TS patients, probably underlying an attempt to accelerate interhemispheric transfer as a compensatory process.

In conclusion, our study confirms that TS is associated with an abnormal ability to lateralize fine motor tasks, which persists throughout different age periods and appears dissociated from the transcallosal connectivity of motor cortical regions. Our findings also suggest that the altered interhemispheric transfer of motor abilities in TS may be the result of compensatory processes linked to self-regulation of motor control. Further research is needed to evaluate whether this trait influences the performance on a wider range of manual skills deployed in everyday life.
